# Application of Classroom Assessment Techniques in Medical Education: Results From the Community Medicine Teaching and Learning Experience

**DOI:** 10.7759/cureus.58930

**Published:** 2024-04-24

**Authors:** Saptarishi Bose, Jyotchna D Bade, M. Balachandra R Naidu, Paromita Roy, Venkataramana Kandi

**Affiliations:** 1 Community Medicine, Great Eastern Medical School and Hospital, Srikakulam, IND; 2 Biochemistry, Great Eastern Medical School and Hospital, Srikakulam, IND; 3 Dentistry, Great Eastern Medical School and Hospital, Srikakulam, IND; 4 Clinical Microbiology, Prathima Institute of Medical Sciences, Karimnagar, IND

**Keywords:** muddiest point, one-minute paper, teaching and learning (tl), exam outcomes, student learning, community medicine, medical education, classroom assessment techniques (cats)

## Abstract

Introduction

Efficient delivery of medical education (ME) is crucial to improving the standards of future physicians or clinicians. India has been experiencing an enormous increase in medical colleges and student admissions into medicine. This has resulted in overcrowding and compromised the student-to-teacher ratio. Conversely, students and teachers face difficulties with learning and teaching, respectively. Classroom assessment techniques (CATs) offer an egalitarian and productive approach to student learning and evaluation. This study was conducted to understand the role of CATs in improving student learning and motivation during community medicine lectures. Further, this study assessed the classroom teaching and learning (TL) process.

Method

This study included 100 third-year medical students pursuing a Bachelor of Medicine and Bachelor of Surgery (MBBS) and 12 faculty members working at Great Eastern Medical School and Hospital (GEMS&H), Srikakulam, India. To facilitate learning and boost motivation, this study applied three CATs including a one-minute paper (OMP), muddiest point (MP), and student-generated test questions (SGTQs). After two months of applying CATs, the teachers and students were asked for feedback on their experiences. The data generated from feedback forms were tabulated and analyzed.

Results

According to 76% (76/100) of students, these strategies have stimulated their interest in learning community medicine. Besides, 64% (64/100) of students believed utilizing these strategies would improve their exam outcomes. Further, 77% (77/100) of students believed these methods must be applied in subsequent lessons. About 68% (68/100) of students thought other subject teachers should also employ these strategies. Of the 12 faculty members included in the study, they mostly liked the OMP (5; 41.66%) and MP technique (5; 41.66%).

Conclusions

Teachers and students have highly welcomed the utility of CATs to improve learning in community medicine. Of the three CATs applied, the OMP was the most popular with students, and teachers agreed that using OMP in the classroom along with MP would be beneficial. Most students and teachers were enthusiastic about employing additional TL strategies like CATs.

## Introduction

Medical education (ME) involves training medical students pursuing a Bachelor of Medicine and Bachelor of Surgery (MBBS). MBBS students undergo training in multiple but interrelated subjects throughout the course [1]. This is associated with complexities in teaching and learning (TL) processes. Therefore, efficient curriculum delivery depends on the strategies applied to the TL process. This greatly affects the training and skills developed by the students allowing them to become better physicians or clinicians.

In the era of digitalization and the internet, classroom TL processes have been greatly influenced by the ME strategies utilized. This is evident because medical subjects are complex and cannot be completely understood by conventional modes like regular/conventional classroom lectures. Many interventions adjunct to didactic/monotonous lectures like group discussions, solving multiple choice questions (MCQs) individually and in combination, and problem-based learning to teach medical subjects have been suggested [2,3].

Most ME strategies are focused on ways to improve student's learning in the classroom. Recently, teachers' expanded and comprehensive use of assessment inside the classroom is particularly gaining importance. In this process, the teachers keep track of the student learning process during classroom teaching and use this information to make necessary amendments for future lectures.

The day-to-day learning process of the students in the classroom is assessed based on a formative assessment test (FAT). Formative evaluation is an important tool regularly used to evaluate the training of medical students [4]. If conducted appropriately and regularly, the teachers can ensure increased learning and higher comprehension in students. FAT can yield useful information while assessing the effectiveness of instructional methods and student learning [5]. Despite its usefulness, routinely employed FAT may not be the right choice to evaluate the TL process that happens in the classroom.

Simple, non-graded in-class exercises called classroom assessment techniques (CATs) are created to provide students and educators with helpful and rapid input on the TL process [6,7]. CATs use a formative rather than summative method of evaluation. The classroom evaluation allows teachers and students to enhance the effectiveness of the TL process. The purpose of classroom assessments is to notify teachers in real-time, about what, how much, and how effectively students are learning.

Additionally, such evaluations can be created to encourage writing or critical thinking abilities and boost student motivation to take themselves and their education more seriously. The teachers can use different CATs before, during, or after a lecture using many methods. These methods can test students' ability to recall information, comprehend it, use creativity and critical thought, and apply those skills practically. The choice of technique utilized depends on the logistics, available time, feasibility, and depends on the subject. These methods help the students to develop higher-order thinking, such as knowledge synthesis, problem-solving, and understanding how to learn [8].

Given the significance of daily assessment of classroom learning among the students and the need for constructive feedback on the TL process, CATs are recommended. Employing CATs enables evaluation of the level of preparation by the teachers. This study tried to analyze the effectiveness of classroom teaching, students' and teachers' perceptions of CATs, and their impact on the TL process.

## Materials and methods

This descriptive study involved 100 MBBS students in their third year of study, and 12 faculty members. The study was conducted in the Department of Community Medicine at Great Eastern Medical School and Hospital (GEMS&H), Srikakulam, India. All participants in the study gave their consent and were informed of the study's goals and methodology. Ethical clearance was obtained from the Institutional Ethics Committee of GEMS&H (05/IEC/GEMS&H/2022). The teaching faculty of the Department of Community Medicine was apprised of the introduction of CATs. Three CATs (one-minute paper (OMP), muddiest point (MP), and student-generated test questions (SGTQs)) were introduced in lectures by the teachers for two months. A three-point Likert scale was used to evaluate the questionnaire filed by students. The teachers chose the technique randomly and the choice was based on the topic discussed in the classroom.

Brief description of CATs

There are different CATs available to use by teachers. The background knowledge probe, the OMP, MP, what’s the principle?, and the defining features matrix are some CATs proposed to enhance the TL process [7]. In this study, we have employed OMP, MP, and SGTQs.

The One-Minute Paper

OMP is a flexible evaluation method, used in classrooms for quick and easy feedback [9]. The teacher finishes the class ten minutes early and requests the students to write briefly about the following questions in one minute: “What was the most significant lesson you learned in this class?” They were instructed to write their responses in either words, phrases, or brief sentences. This method aids in determining the pupils' degree of prior knowledge, recall, and comprehension.

The Muddiest Point

The instructor requests a quick response from the class with the following query: "What was the MP (most confusing) in today's lecture?" [10]. This method provides direct feedback from the students on the concepts they find least understandable. The faculty will use this information to determine what to emphasize and how much time to allot for similar issues to the upcoming topics.

Student-Generated Test Questions

This task requires the students to create two or more original test questions based on the lecture topic. Students had to complete this exercise either individually or in groups. The questions may be of short answer or multiple choice type. The students must know the solution to the questions they have framed. A quiz shall be conducted using the student-prepared questionnaire. If the class fails to answer a question during the quiz, the group or individual presenting will explain the answer.

Students’ Response

At the beginning of the class, the students were told about the techniques to be used. They were asked not to write their names on the response papers, which allowed them to write their responses without hesitation. The responses were collected and the teacher addressed the most frequent responses to the students in the next class within the same week.

Collection of Feedback

After utilizing CATs for around two months, faculty and students were asked to provide feedback on the utility of these strategies in the regular classroom setting.

Student Feedback

Students were given a feedback form and asked for responses based on their perception of the implementation of CATs on a three-point Likert scale.

Faculty Feedback

**Figure 1:**
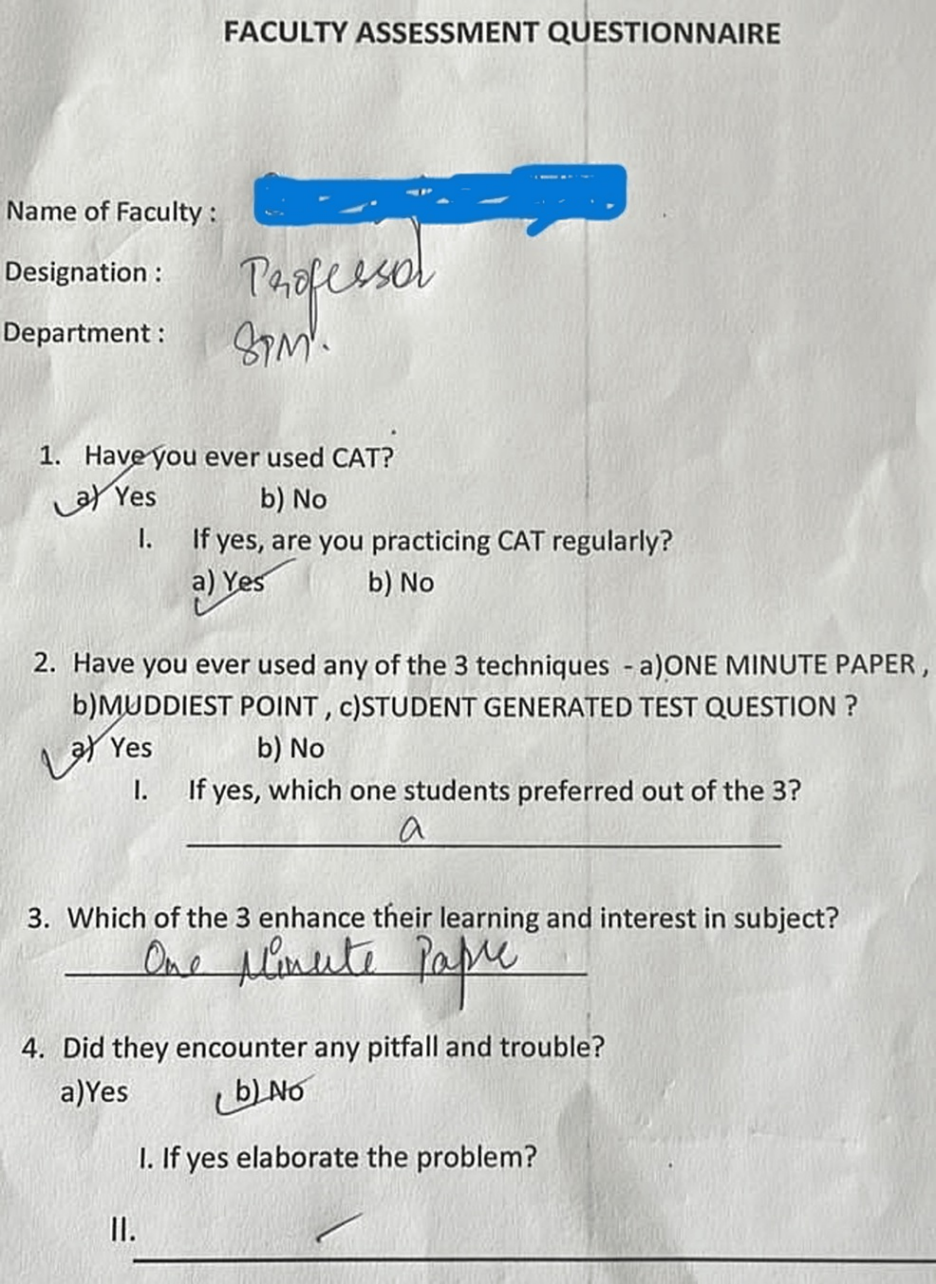
Faculty feedback questionnaire CATs: Classroom assessment techniques

An open-ended questionnaire was used to collect feedback from the faculty (Figure 1).

Statistical analysis

The data obtained were entered into a Microsoft Office 2019 Excel sheet (Microsoft® Corp., Redmond, United States), and statistical inferences were drawn using IBM SPSS Statistics for Windows, Version 20 (Released 2011; IBM Corp., Armonk, New York, United States). The quantitative data were represented as percentages.

## Results

Of the total 100 students included in the study, 47 (47%) were females and 53 (53%) were males. Cumulatively, 67.87% of the students felt that the CATs and similar strategies positively impacted the TL processes. About 70% of the students believe that CATs improved their interest in community medicine. A majority of the students noted that the use of an OMP enhanced their learning and interest in the subject. The details of students' perceptions of various aspects of CATs are shown in Table 1.

**Table 1:** Student's perception of CATs CATs: Classroom assessment techniques

Question	Disagree n = 100; n%	Neutral n = 100; n%	Agree n = 100; n%
CATs enhanced my learning in Community Medicine	8	33	59
CATs enhanced my interest in Community Medicine	9	15	76
The use of a one-minute paper helped to enhance my learning and interest	5	15	80
The use of the muddiest point helped to improve my learning and interest	13	24	63
The use of student-generated test questions enhanced my learning and interest	17	27	56
The use of these techniques during classes will affect my performance in the examinations positively	10	26	64
Similar strategies should be used in future classes	5	18	77
Other subject teachers should use CATs and similar strategies	9	23	68
Average (%)	8.87	22.66	67.87

**Figure 2:**
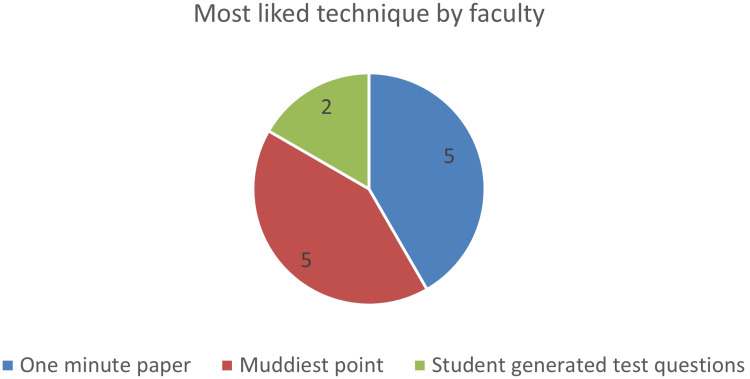
Image depicting most liked techniques among the faculty

Of the 12 faculty members included in the study, the teachers mostly liked the OMP (5; 41.66%) and MP technique (5; 41.66%) as shown in Figure 2.

## Discussion

ME assumes greater significance in the context of patient management. MBBS graduates are expected to function as physicians of first contact. However, recently the standard of ME in countries like India has been in question. This could be attributed to the increased number of medical colleges, faculty deficiencies, infrastructural limitations, and other factors [1].

Students undergo periodic evaluations, including theoretical and practical examinations, including written tests (like essay questions, short answer questions, MCQs, etc.), oral exams (like vivavoce), objective structured clinical examination (OSCE), and objective structured practical examination (OSPE) [11-13]. However, these examinations do not reflect the quality of the TL process during lectures. A recent study suggested the introduction of a student-centered formative assessment in medical curricula [14].

Particularly, the classroom TL process has been severely affected by the increased intake of students in the MBBS course. A modern classroom may have around 200 medical students, significantly influencing the TL process. Despite the use of advanced technologies like audio-visual aids including PowerPoint projections and large screens in the classrooms, there could be difficulties in understanding the topic from the students' point of view. Besides, the huge crowd leaves the teachers unaware of whether the students have been able to comprehend the subject of the lecture.

The evaluation process in an MBBS course in India happens every 3-4 months in the form of theory and practical examinations. The student performance in these examinations does not reflect the effective TL process in the classrooms. This leaves a significant void in the responsibility of the teachers and students in the classroom.

CATs are easy tools to measure how well the students are involved in the TL process [5,6]. The teacher can assess the student's comprehension of the topic in the classroom. This allows the teachers to get immediate feedback from the students. Teachers can understand the areas of difficulties, that help them overcome those in the next lecture. The desire and motivation to learn more about the subject grows in students.

According to a study by Zhao et al. from China, CATs were simple to use in the primary school classroom and provided teachers with new insights into the students' learning. Teachers expressed their enthusiasm to implement CATs as a part of TL activities in the classroom [15]. A previous study proposed that CATs encourage self-assessment by the student and reflections among both the teachers and students [16]. However, care must be taken in choosing appropriate CATs and allowing enough time in class to ensure they are beneficial.

Despite their potential benefits, CATs have rarely been tested/utilized/experimented by medical educators. Kumar et al. in their study introduced CATs into teaching biochemistry to first-year MBBS students. Feedback from this study revealed that 85.7% of faculty and 75.2% of students are very positive and looking forward to using CATs in the future. Further, 73.3% of students felt these techniques would enhance learning and motivation. Like the current study results, 71.4% of faculty and 67.6% of students felt other subject teachers should also start using such techniques [17].

A study by Srivastava et al. from Maharashtra, India, which included 100 first-year medical students, conducted a case-control study. This study evaluated the efficacy of CATs like classroom quizzes, exit slips, OMP, MP, logic model, one-sentence summary, and directed paraphrasing. The results of this study noted that OMP and MP activated cognitive thinking, contributed to self-assessment, and identified knowledge gaps [18].

OMP was identified as a cost-effective and easy classroom assessment tool that facilitates student participation in classroom lectures. OMP allows students to self-assess, provide feedback, and raise queries regarding the lecture topic [19].

In the present study, out of the three strategies used in the lectures, OMP was the most popular (80%) among students. Most teachers agreed that using OMP in the classroom along with MP would be beneficial. The MP assessment technique can promote declarative learning and facilitate recall and understanding. It was proposed that MP has an immediate positive impact on improving the TL process [5].

Study limitations

This study has been conducted in one of the departments of a tertiary care teaching hospital and involves MBBS students in their third year of study. Further large-scale studies involving many students at different stages of MBBS and teachers of different faculties/subjects could be required to provide additional clear evidence of the utility of CATs for ME.

## Conclusions

Through this study, we introduced the CATs to teach the subject of community medicine to third-year MBBS students. The results stand as a piece of evidence that students have piqued their interest in learning community medicine. It was also evident that these strategies could improve student's exam performance and outcomes. Many students felt these methods must be applied in subsequent lectures. Further, students thought other subject teachers should also employ these strategies. A majority of students suggested that CATs helped them retain the subject of community medicine, thereby enhancing their interest in learning this subject. Based on the results of this study, CATs are recommended to be included in ME strategies to improve the TL process.

## References

[1] Kandi V (2022). Medical education and research in India: a teacher's perspective. Cureus.

[2] Vadakedath S, Kandi V (2019). Modified conventional teaching: an assessment of clinical biochemistry learning process among medical undergraduate students using the traditional teaching in combination with group discussion. Cureus.

[3] Kandi V, Basireddy PR (2018). Creating a student-centered learning environment: implementation of problem-based learning to teach microbiology to undergraduate medical students. Cureus.

[4] Almahal EA, Osman AA, Tahir ME, Hamdan HZ, Gaddal AY, Alkhidir OT, Gasmalla HE (2023). Fostering formative assessment: teachers' perception, practice and challenges of implementation in four Sudanese medical schools, a mixed-method study. BMC Med Educ.

[5] Melland HI, Volden CM (1998). Classroom assessment: linking teaching and learning. J Nurs Educ.

[6] Angelo TA, Cross KP (1993). Classroom Assessment Techniques. Handbook for College Teachers. https://smartlib.umri.ac.id/assets/uploads/files/456bd-classrooms-assessement-techniques.pdf.

[7] (2024). Classroom assessment techniques (CATs). https://cft.vanderbilt.edu/guides-sub-pages/cats/.

[8] Davidson JE (2009). Preceptor use of classroom assessment techniques to stimulate higher-order thinking in the clinical setting. J Contin Educ Nurs.

[9] Ashakiran S, Deepthi R (2013). One-minute paper: a thinking centered assessment tool. Internet J Med Update.

[10] (2024). Muddiest point in the lecture (muddy cards). http://www.cdio.org/files/mudcards.pdf.

[11] Elshama SS (2020). How to use and apply assessment tools in medical education?. Iberoam J Med.

[12] Kipkulei J, Kangethe S, Boibanda F, Jepngetich H, Lotodo T, Kirinyet J (2022). Assessment methods used during clinical years of undergraduate medical education at Moi university school of medicine, Kenya. Health.

[13] Sheriff DS (2023). Assessment methods in medical education. Ann SBV.

[14] Ma T, Li Y, Yuan H (2023). Reflection on the teaching of student-centred formative assessment in medical curricula: an investigation from the perspective of medical students. BMC Med Educ.

[15] Zhao X, Van den Heuvel-Panhuizen M, Veldhuis M (2016). Teachers’ use of classroom assessment techniques in primary mathematics education-an explorative study with six Chinese teachers. IJ STEM Ed.

[16] Walker DM (2012). Classroom assessment techniques: an assessment and student evaluation method. Creat Educ.

[17] Kumar A, Kumar MS, Gulia R (2017). The use of classroom assessment techniques (CATs) to enhance learning and motivation in first year undergraduate students. J Res Med Edu Ethics.

[18] Srivastava TK, MIshra V, Waghmare LS (2018). Formative assessment classroom techniques (FACTs) for better learning in pre-clinical medical education: a controlled trial. J Clin Diagn Res.

[19] Sahoo BK, Taywade M (2021). One minute paper: the reflective way to teach and learn in medical education. Indian J Forensic Community Med.

